# Reversal of the Apoptotic Resistance of Non-Small-Cell Lung Carcinoma towards TRAIL by Natural Product Toosendanin

**DOI:** 10.1038/srep42748

**Published:** 2017-02-17

**Authors:** Xin Li, Ming You, Yong-jian Liu, Lin Ma, Pei-pei Jin, Ri Zhou, Zhao-Xin Zhang, Baojin Hua, Xiao-jun Ji, Xiao-ying Cheng, Fangzhou Yin, Yan Chen, Wu Yin

**Affiliations:** 1 The State Key Lab of Pharmaceutical Biotechnology, College of life Sciences, Nanjing University, Nanjing, 210093, China; 2 Jiangsu Key Lab of Pediatric Respiratory Disease, Nanjing University of Chinese Medicine, Nanjing, China; 3 The State Key Laboratory of Pharmaceutical Biotechnology, Division of Immunology, Medical School, Nanjing University, Nanjing, China; 4 Department of Anesthesiology and Intensive Care Unit, Changhai Hospital, Affiliated Hospital of the Second Military Medical University, Shanghai, China; 5 College of Pharmacy, Nanjing University of Chinese medicine, China; 6 Guang’anmen hospital, China Academy of Chinese Medical Sciences, Beijing, China; 7 Jiangsu Cancer Hospital & Institute Affiliated to Nanjing Medical University, China

## Abstract

Tumor necrosis factor-related apoptosis-inducing ligand (TRAIL) selectively triggers cancer cell death via its association with death receptors on the cell membrane, but exerts negligible side effects on normal cells. However, some non-small-cell lung carcinoma (NSCLC) patients exhibited resistance to TRAIL treatment in clinical trials, and the mechanism varies. In this study, we described for the first time that toosendanin (TSN), a triterpenoid derivative used in Chinese medicine for pain management, could significantly sensitize human primary NSCLC cells or NSCLC cell lines to TRAIL-mediated apoptosis both *in vitro* and *in vivo*, while showing low toxicity against human primary cells or tissues. The underlying apoptotic mechanisms involved upregulation of death receptor 5 (DR5) and CCAAT/enhancer binding protein homologous protein, which is related to the endoplasmic reticulum stress response, and is further associated with reactive oxygen species generation and Ca^2+^ accumulation. Surprisingly, TSN also induced autophagy in NSCLC cells, which recruited membrane DR5, and subsequently antagonized the apoptosis-sensitizing effect of TSN. Taken together, TSN can be used to sensitize tumors and the combination of TRAIL and TSN may represent a useful strategy for NSCLC therapy; moreover, autophagy serves as an important drug resistance mechanism for TSN.

Lung carcinoma is one of the most common causes of cancer-related death worldwide^1^. Only 17.4% of all lung cancer patients live for 5 years or more after diagnosis^2^. It is estimated that 85% cases of lung carcinoma belong to non-small-cell lung carcinoma (NSCLC). In 2012, 1.8 million people had lung cancer, which resulted in 1.6 million deaths worldwide^3^. Surgery remains the most appropriate treatment choice for patients with stage I or II NSCLC^2^. However, surgery is seldom performed on patients with unresectable stage III or IV NSCLC. For this group of patients, adjunct chemotherapy can improve the 5-year survival rate and prevent recurrence. Currently, many chemotherapeutic agents are available for the treatment of metastatic NSCLC. Among them, platinum compounds (cisplatin or carboplatin) with gemcitabine, vinorelbine, or taxanes are reference regimens^4^. Unfortunately, although these drugs could be used to effectively eradicate tumor cells, most of them inevitably lead to non-specific toxic effects on normal cells or tissues. Thus, there has been an emphasis on the development of anti-cancer therapeutics with a selective killing effect on cancerous cells.

Tumor necrosis factor (TNF)-related apoptosis-inducing ligand (TRAIL), also known as APO-2 ligand and TNFSF10, is a promising anti-cancer agent that belongs to the TNF superfamily. It has been tested in phase II clinical trials, showing selective killing effects on tumors with negligible cytotoxicity against normal tissues^5^. TRAIL exerts its pro-apoptotic effect on tumor cells through association with membrane receptors including death receptor TRAIL-R1 (DR4) and TRAIL-R2 (DR5), whereas the binding of TRAIL to decoy receptors TRAIL-R3 (DcR1), TRAIL-R4 (DcR2), or osteoprotegerin achieves the opposite effect^6^. The interaction of TRAIL and death receptors results in the activation of the initiator caspase-8 and recruitment of Fas-associated protein with death domain (FADD), leading to the formation of death-inducing signaling complex (DISC)^7^. In the wake of activation of caspase-3 by caspase-8, apoptosis occurred in TRAIL-treated tumor cells. Nevertheless, ample evidence suggests that NSCLC cells are relatively insensitive to TRAIL treatment^8^, and the resistance mechanisms include distribution of decoy receptors^9^, persistent activation of AKT and NF-κB, universal upregulation of anti-apoptotic proteins, and mutation of Bax and BAK genes^10^. Hence, increasing the NSCLC cells’ susceptibility to TRAIL has been an important concern in targeted therapy for NSCLC.

The fruit of *MeliatoosendanSieb et Zucc*, also known as Chuan Lian-Zi, is a traditional medicine that has significant analgesic effects; in addition, it can be used to kill parasites such as roundworms^11^. Toosendanin (TSN) is the main active component of the fruit of *MeliatoosendanSieb et Zucc*; it can selectively block acetylcholine (Ach) release and has antibotulismic effects both *in vivo* and *in vitro*^12^. Notably, TSN was recently found to have anti-cancer effects on human cancer cells including PC3, PC12, BEL7404, U251, SHSY5Y, HL60, and U937 cells^13^, suggesting a novel application of TSN in cancer therapy. However, the concentration of TSN used in most studies is relatively high, which increases the risk of non-specific toxicity against normal human cells or tissues, thereby limiting its potential application in clinical cancer treatment.

In the present investigation, we demonstrated for the first time that TSN can significantly reverse the resistance of NSCLC to TRAIL-induced apoptosis both *in vivo* and *in vitro*. Furthermore, the concentration of TSN used was very low to cause toxic side effects in normal cells or tissues. The endoplasmic reticulum (ER) stress response plays a critical role in cancer cell survival, apoptosis, and drug resistance. In this study, we found that the sensitizing effect of TSN was largely dependent on the ER stress response, but interestingly, while TSN reversed NSCLC resistance, NSCLC cells in turn triggered autophagy to counteract the effect of TSN, thereby constituting an important drug resistance mechanism for TSN. In summary, TSN can be used as a pharmacological agent to sensitize NSCLC to TRAIL-mediated apoptosis. Additionally, understanding the drug resistance mechanism of TSN is very useful for determining its potential application in cancer treatment.

## Results

### TSN selectively increases sensitivity of NSCLC cells, but not normal cells, to TRAIL-induced apoptosis

**Figure 1:**
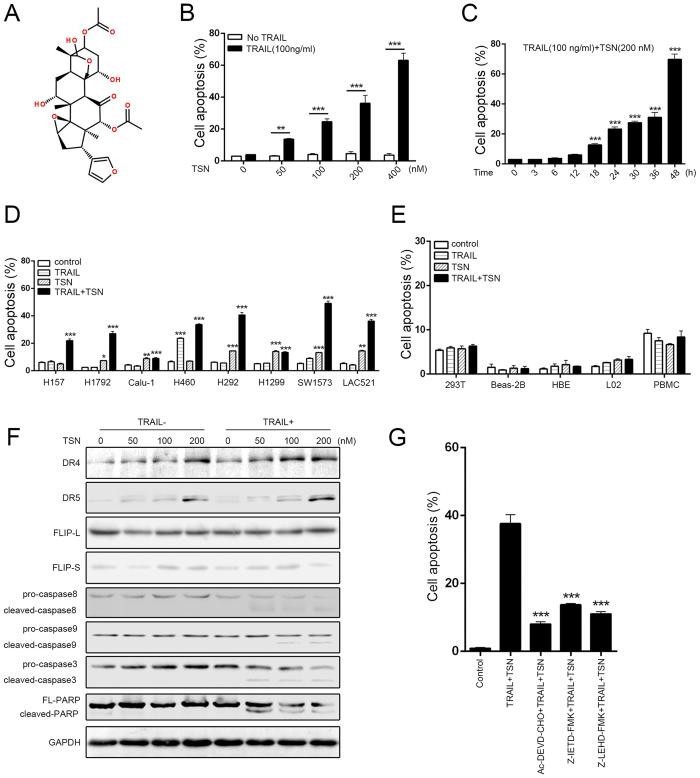
TSN reverses the resistance of NSCLC cells to TRAIL-mediated apoptosis. (**A**) Chemical structure of toosendanin. (**B**) A549 cells were treated TSN, TRAIL, or both for 24 h, after treatment, cell apoptosis was measured by annexin V/PI double staining method. The concentrations for TSN used were 0, 50, 100, 200, 400 nM, respectively. TRAIL was used at a fixed concentration of 100 ng/ml. Data represent the mean ± SEM from three independent experiments, ***P < 0.001, **P < 0.01, TSN alone vs. TRAIL + TSN. (**C**) A549 cells were incubated with 100 ng/ml TRAIL and 200 nM TSN for indicated time. After treatment, cell apoptosis was measured by flow cytometry. Data represent the mean ± SEM from three independent experiments, ***P < 0.001, **P < 0.01, as compared with DMSO-treated cells (0 h). (**D**) NSCLC cell lines and primary tumor cells isolated from clinical samples (lung adenocarcinoma 521, LAC521) were treated with TRAIL (100 ng/ml), TSN (100 ng/ml), or both. The proportion of apoptotic cells was measured at 24 h after treatment by flow cytometry. Data represent the mean ± SEM from three independent experiments, ***P < 0.001, **P < 0.01, *P < 0.05, as compared with control. (**E**) Normal cell lines 293 T, BEAS-2B, HBE, L02, and PBMC were subjected to the same treatment as in (**D**); the proportion of apoptotic cells was measured by flow cytometry. Data represent the mean ± SEM from three independent experiments. (**F**) A549 cells were treated with the indicated concentrations of drugs, after treatment, western blot analysis was performed to measure the protein expression levels. GAPDH was included as a loading control; each experiment was performed at least in triplicate. (**G**) A549 cells were pre-incubated with caspase inhibitors (5 μM) for 30 min, then treated with TRAIL (100 ng/ml) and TSN (200 nM) for 24 h. Cell apoptosis was measured by annexin V/PI staining. Data represent the mean ± SEM from three independent experiments, ***P < 0.001, as compared with cells treated with TRAIL plus TSN.

Previous studies and clinical trials showed that some NSCLC cells are relatively resistant to TRAIL therapy^8^. In this study, we first investigated the apoptotic effects of TRAIL and TSN on A549 cells because this cell line is relatively TRAIL-resistant. The chemical structure of TSN is shown in Fig. 1A. Treatment with TRAIL or TSN alone did not affect A549 cells viability, but when A549 cells were treated with a combination of TRAIL and TSN, a significant increase in apoptosis occurred in a dose- and time-dependent manner (Fig. 1B and C). To further confirm this effect, other NSCLC cell lines were used (Fig. 1D). Among these cell lines, H157, H1792, Calu-1, H292, H1299, and SW1573 are TRAIL resistant, but H460 cells are TRAIL sensitive. The results demonstrated that the combination of TSN and TRAIL significantly increased the percentage of apoptotic cells in both TRAIL-resistant cells (H157, H1792, H292, and SW1573) and TRAIL-sensitive cells (H460). Notably, LAC521 cells are primary NSCLC cells that were isolated from NSCLC patients in our lab; they are TRAIL-resistant because no significant cell apoptosis occurred in these cells after TRAIL treatment. However, TSN can significantly increase the sensitivity of LAC521 primary NSCLC cells to TRAIL-induced apoptosis. To evaluate whether the combined use of TSN and TRAIL has a toxic effect on normal cells, human peripheral blood mononuclear cells (PBMCs) isolated from fresh blood and human cell lines including 293T, Beas-2B, HBE, and L02 were examined. The results showed that the combined use of TRAIL and TSN did not trigger significant cell apoptosis in these cells (Fig. 1E), indicating that the effect of TSN is tumor cell-specific.

Caspase activation is a hallmark of cell apoptosis. In this study, we found that TRAIL or TSN alone failed to activate the cleavage activity of pro-caspase 9, pro-caspase 8, and pro-caspase 3; however, the cleavage bands of caspase 3, caspase 8, caspase9, and PARP were evident in cells after the combined use of TRAIL and TSN (Fig. 1F). Both DR4 and DR5 are TRAIL receptors. In this study, TSN alone dose-dependently increased the protein expression of DR4 and DR5. Notably, TSN alone failed to increase membrane DcR1 and DcR2 abundance, as examined by flow cytometry (Supplementary Fig. S1). FLIP-L and FLIP-S are anti-apoptotic proteins that can block death receptor-mediated apoptotic signaling; we found that TSN failed to alter the expression of these proteins, regardless of whether TRAIL was present or absent. To further confirm the involvement of caspase activation in cell apoptosis, pharmacological inhibitors were used. The results showed that caspase 3, caspase 8, and caspase 9 inhibitors suppressed the cell apoptosis induced by TRAIL plus TSN (Fig. 1G).

### Role of TRAIL death receptors in the apoptosis-enhancing effect of TSN

**Figure 2:**
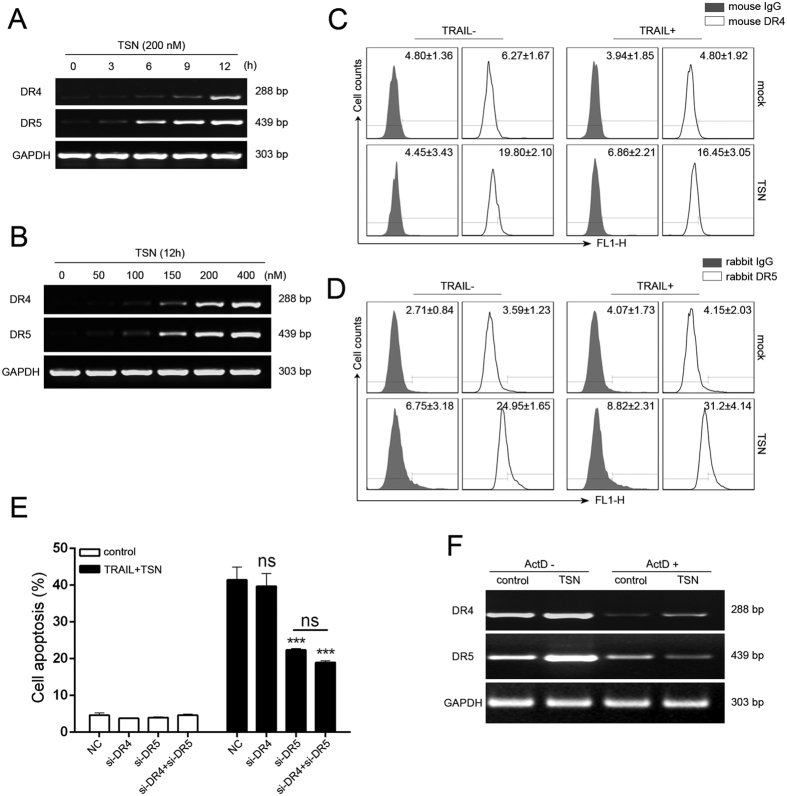
Involvement of death receptors in TSN-mediated apoptosis enhancement. (**A**) A549 cells were treated with 200 nM TSN for indicated time. After treatment, RT-PCR analysis was performed to measure the expression of DR4 and DR5 at mRNA levels. GAPDH was included as a loading control; each experiment was performed at least in triplicate. (**B**) A549 cells were treated with TSN at increasing concentrations for 12 h. After treatment, mRNA levels of death receptors were assessed by RT-PCR analysis. GAPDH was included as a loading control; each experiment was performed at least in triplicate. (**C**,**D**) A549 cells were treated with 200 nM TSN for 12 h, cell surface expression levels of death receptor 4 (**C**) or death receptor 5 (**D**) were measured by flow cytometry. Each experiment was performed at least in triplicate. (**E**) A549 cells were transfected with siRNAs against DR4, DR5, or both for 24 h before drug treatments. After 24 h drug treatment, cells were collected and apoptosis was measured. Data represent the mean ± SEM from three independent experiments, ***P < 0.001, as compared with cells transfected with siRNA control. (**F**) A549 cells were pretreated with Act D at 5 ug/ml for 30 min, and then subjected to 200 nM TSN treatment for 12 h. RT-PCR analysis was performed to assess death receptors 4/5 expression. GAPDH was included as a loading control; each experiment was performed at least in triplicate.

It has been well-established that TRAIL-induced cell apoptosis is mediated by its interaction with DR4 and DR5 receptors^14^. In the present study, TSN not only increased DR4 and DR5 protein expression as shown above, but also time- and dose-dependently increased DR4 and DR5 gene transcription (Fig. 2A and B). Because transportation of death receptors from cytosol to cell surface is critical for TRAIL-induced apoptosis^15^, we measured the surface expression levels of DR4 and DR5 by flow cytometry. As shown in Fig. 2C and D, TSN treatment alone significantly increased the abundance of DR4 and DR5 on cell membranes. To analyze whether the upregulation of DR4 and DR5 is a side effect, or contrarily, necessary for TSN-mediated sensitization to TRAIL-induced apoptosis, we used siRNA technology to knock down the expression of DR4 and DR5 in A549 cells (supplementary result Fig. S2). The results demonstrated that siRNA-mediated DR4 suppression failed to decrease the TSN/TRAIL-induced cell apoptosis, while blockage of DR5 expression significantly reduced the rate of cell apoptosis (Fig. 2E). In addition, there was no additive effect when both DR4 and DR5 expression was silenced. These data provide evidence that DR5 plays a crucial role in the restoration of TRAIL sensitivity by TSN. Because TSN can upregulate DR4 and DR5 expression at both mRNA and protein levels, we introduced actinomycin D (Act D) to determine at which stage this upregulation occurred. The results showed that Act D suppressed TSN-mediated upregulation of death receptors at the mRNA level (Fig. 2F), indicating that TSN regulates DR4/5 gene expression mainly through transcription.

### TSN increases CHOP-mediated DR5 transcription

**Figure 3:**
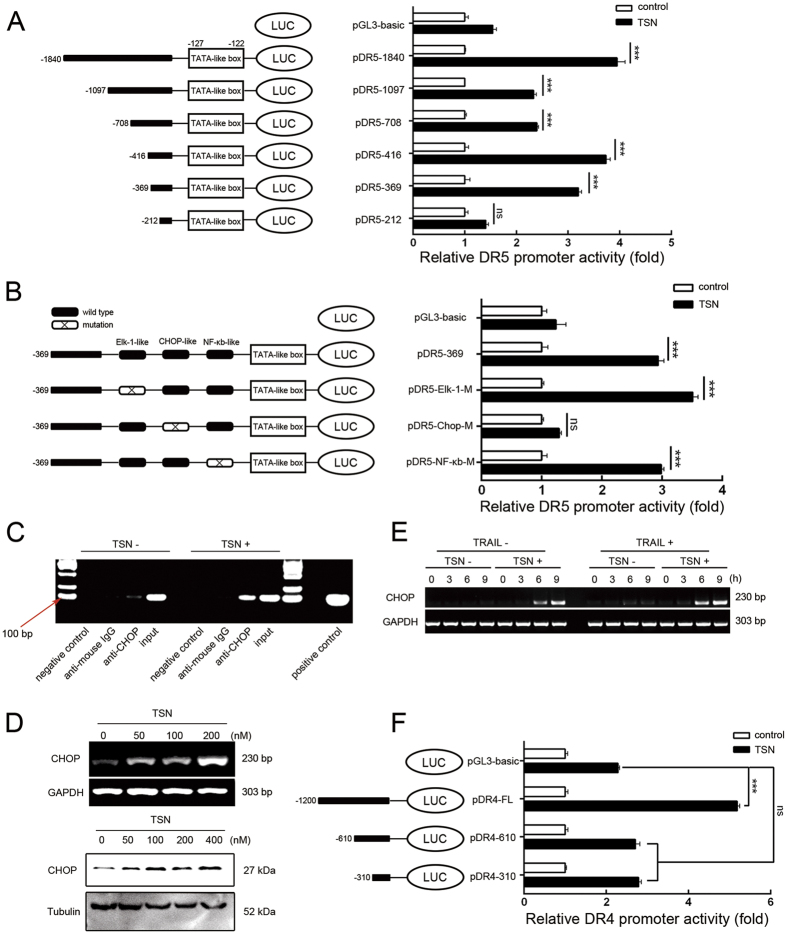
TSN upregulates the expression of death receptors at transcriptional levels. (**A**) Construction of DR5 promoter reporter plasmid and its deletion mutants (left panel). A549 cells were transiently transfected with 0.8 μg indicated constructs and 0.2 μg pRL-TK for 24 h, and then treated with or without 200 nM TSN for another 24 h (right panel). The luciferase activity of untreated samples was arbitrarily set at 1.0 in each group. Data represent the mean ± SEM from three independent experiments. ***P < 0.001, as compared with control in each group. (**B**) Construction of DR5 promoter variants with point mutation in indicated sites (Left panel). A549 cells were transiently transfected with 0.8 μg indicated constructs and 0.2 μg pRL-TK for 24 h, and then treated with or without 200 nM TSN for another 24 h (right panel). The luciferase activity of untreated samples was arbitrarily set at 1.0 in each group. Data represent the mean ± SEM from three independent experiments. ***P < 0.001, as compared with control in each group. (**C**) A549 cells were challenged with 200 nM TSN for 6 h, binding of CHOP with DR5 promoter was assessed in a ChIP assay following the protocols described in materials and methods. IgG was used as a non-specific control. Each experiment was performed at least in triplicate. (**D**) A549 cells were treated with the indicated concentrations of TSN. After treatment, RT-PCR and western blot analyses were performed to evaluate the expression levels of CHOP. GAPDH and tubulin were used as loading controls; each experiment was performed at least in triplicate. (**E**) A549 cells were incubated with 200 nM TSN or 100 ng/ml TRAIL or both for indicated time. The mRNA expression level of CHOP was measured by RT-PCR analysis. GAPDH and tubulin were used as loading controls; each experiment was performed at least in triplicate. (**F**) Construction of DR4 promoter reporter plasmid and its deletion mutants (left panel). Drug treatment was similar as that in (**A**). Data represent the mean ± SEM from three independent experiments. ***P < 0.001, as compared with control in each group.

To further determine how TSN increases DR4/5 transcription, we constructed luciferase reporters containing different lengths of DR5 promoter region; the construction map is shown in Fig. 3A (left panel). TSN significantly increased the luciferase activity of the DR5 reporters, except pDR5-212 (Fig. 3A), indicating that the DR5 promoter region between −212bp and −369bp has essential elements responsible for TSN-induced transactivation. Bioinformatics analysis revealed that the three transcription factor binding sites (TFBS) of Elk-1, CCAAT/enhancer binding protein homologous protein (CHOP), and NF-κB are located in the promoter region of DR5 spanning −212 bp to −369 bp; thus, we mutated these three TFBS to identify the potential cis-element responsible for TSN treatment. As presented in Fig. 3B, TSN-induced increase in luciferase activity was suppressed by mutation of the CHOP binding site, but not the Elk-1 or NF-κB binding site. Chromatin immunoprecipitation (ChIP) analysis further showed the enhanced binding of CHOP with the DR5 promoter after TSN treatment (Fig. 3C). Therefore, the CHOP binding site is critical for DR5 promoter activation by TSN. TSN dose-dependently stimulated CHOP expression at both mRNA and protein levels in A549 cells (Fig. 3D). It appeared that TSN-induced CHOP upregulation occurred independently of TRAIL. TRAIL alone failed to induce CHOP expression; however, TRAIL plus TSN treatment led to higher CHOP expression than that observed with TSN alone (Fig. 3E), suggesting apoptosis occurrence may strengthen the CHOP induction.

Notably, TSN also increased the luciferase activity of the full-length DR4 promoter reporter, but not of pDR4-610 or pDR4-310, suggesting that the region between −1200 bp and −610 bp within the DR4 promoter may harbor TSN-sensitive cis-elements (Fig. 3F). However, as DR4 contributed less to the sensitizing effect of TSN than DR5 did, DR4 transcription was ignored in this study.

### CHOP and ER stress are critical for the sensitizing effect of TSN

**Figure 4:**
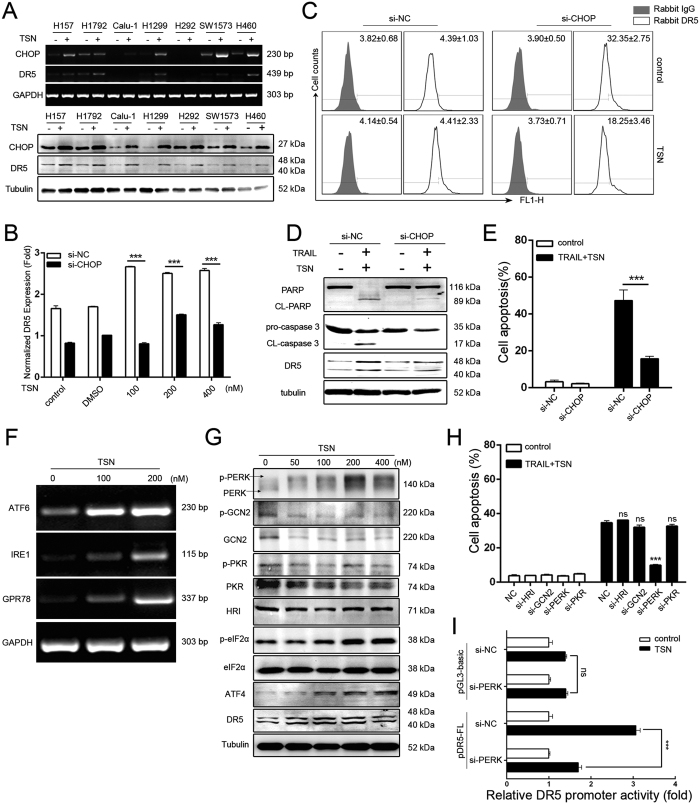
Involvement of CHOP and ER-stress in the effect of TSN. (**A**) NSCLC cells were treated with or without 200 nM TSN for 9 h (upper panel) or 24 h (lower panel). The total cellular mRNAs or proteins were subjected to RT-PCR or western blot analyses. Each experiment was performed at least in triplicate. (**B**) A549 cells were transfected with the indicated siRNAs for 24 h before treatment with TSN for 12 h. qPCR analysis was performed to measure the expression of DR5 at mRNA levels, ***P < 0.001, as compared with cells transfected with si-NC. (**C**) A549 cells were transfected with the indicated siRNAs for 24 h before exposure to the indicated treatment for 12 h, surface expression of death receptors was measured by flow cytometry; each experiment was performed at least in triplicate. (**D**) After transfection with indicated siRNAs, A549 cells were exposed to TRAIL (100 ng/ml) and TSN (200 nM) for another 24 h. The total protein was subjected to western blot analysis; similar results were obtained in three independent experiments. (**E**) After transfection with siRNAs and treatment with TRAIL and TSN, A549 cell apoptosis was measured by flow cytometry, **P < 0.01, as compared with cells treated with cells transfected with si-NC. (**F**) After treatment with different concentrations of TSN for 8 h, A549 cells were lysed and the expression level of ER-stress related genes was assessed by RT-PCR. GAPDH was included as loading control. (**G**) A549 cells were treated with TSN at different concentrations for 24 h before being subjected to western blot analysis for ER-stress-related proteins. (**H**) A549 cells were transiently transfected with the indicated siRNAs for 24 h and exposed to 100 ng/ml TRAIL and 200 nM TSN for another 24 h. The cell apoptosis was measured by flow cytometry, ***P < 0.001, as compared with cells transfected with control siRNA. (**I**) A549 cells were co-transfected with control siRNA/PERK siRNA and DR5 promoter plasmid for 24 h before treatment with or without TSN. The luciferase activity of the untreated samples was arbitrarily set at 1.0 in each group, ***P < 0.001, as compared with control.

CHOP, also known as growth GADD153 or DDIT3, normally acts as a transcription factor. In this study, we found that TSN-mediated CHOP induction is common, because CHOP upregulation (mRNA, top panel; protein, below panel, Fig. 4A) occurred not only in A549 cells, but also in other types of NSCLC cell lines including H157, H1792, Calu-1, H1299, SW1573 and H460 following TSN treatment (Fig. 4A). To further characterize the role of CHOP, CHOP siRNA was employed (supplementary result Fig. S3). The results showed that TSN-induced DR5 protein expression (Fig. 4B) and membrane distribution (Fig. 4C) were largely attenuated in cells when CHOP expression was silenced. As expected, co-treatment of A549 cells with TRAIL and TSN did not achieve a significant apoptotic effect in the presence of CHOP siRNA, as evidenced by the decreased cleavage of PARP and caspase 3 (Fig. 4D). Annexin V(+)/PI(−) apoptotic cells also decreased in si-CHOP transfectant compared to that in mock siRNA transfectant (Fig. 4E).

CHOP upregulation is a critical step for ER stress^16^. Therefore, we examined whether ER stress is involved in TRAIL plus TSN-induced apoptosis. The results showed that the expression of GPR78, along with that of other ER stress sensors including ATF6 and IRE1, sharply increased at the mRNA level following TSN treatment (Fig. 4F). Western blot assay showed that the ATF4 protein level increased following treatment with TSN in a concentration-dependent manner. TSN triggered the phosphorylation of eIF2α, an indicator for general translational arrest. The eIF2α phosphorylation can be triggered by four upstream kinases: HRI, PKR, GCN2, and PERK. In this study, we observed that TSN dose-dependently triggered the phosphorylation of PERK, but not of GCN2, PKR, and HRI, suggesting that PERK is likely responsible for TSN-induced phosphorylation of eIF2α (Fig. 4G). To verify this, we employed siRNA technology to knockdown the expression of these four eIF2α kinases (supplementary result Fig. S4). As expected, the pro-apoptotic effect of TRAIL plus TSN was compromised in cells after transfection with PERK siRNAs, but not after transfection with GCN2, HRI, or PKR siRNAs (Fig. 4H). TSN-induced DR5 transcription was largely attenuated in cells after transfection with PERK siRNAs (Fig. 4I). Therefore, ER stress-mediated CHOP upregulation is critically involved in the sensitizing effect of TSN on TRAIL-mediated apoptosis.

### Involvement of reactive oxygen species generation in TSN-mediated apoptosis enhancement

**Figure 5:**
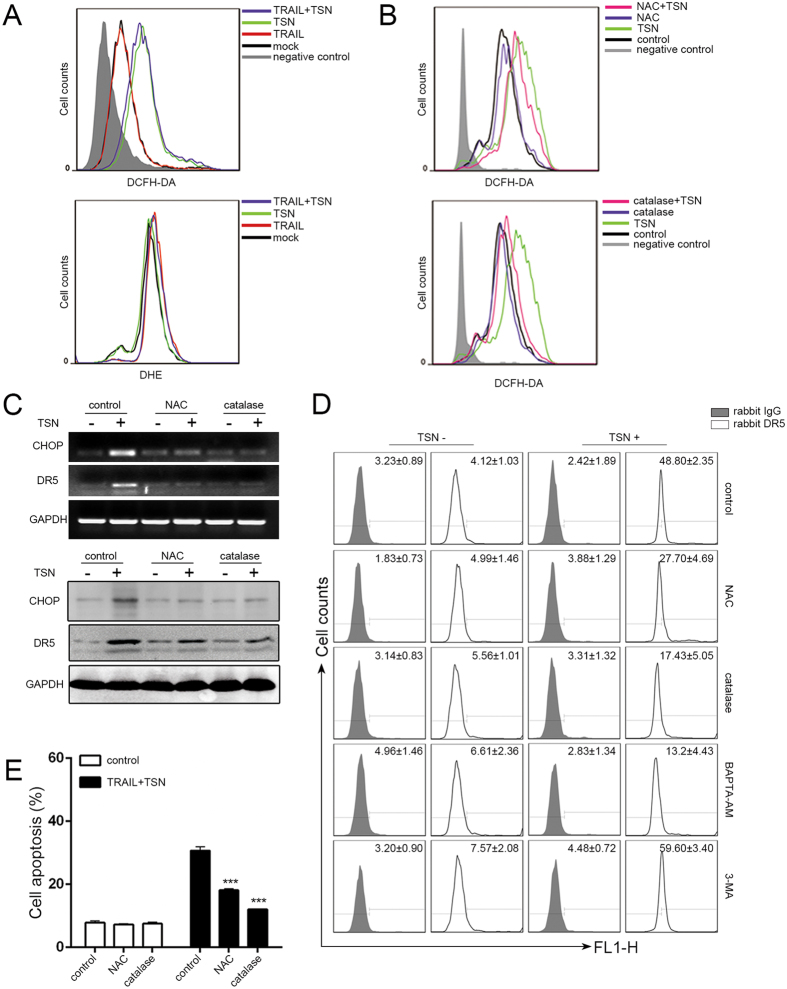
ROS generation is required for TSN-induced CHOP and DR5 upregulation. (**A**) A549 cells were exposed to TSN, TRAIL, or TRAIL + TSN for 12 h, and then cells were collected and stained with DCFH-DA or DHE for measurement of H_2_O_2_, O_2_^−^.,respectively by flow cytometry. Each experiment was performed at least in triplicate. (**B**) Cells were pre-treated with ROS inhibitors (5 mM NAC or 100 U/ml catalase) for 30 min, and then treated with the indicated drugs. After treatment, cells were stained with DCFH-DA for the measurement of H_2_O_2_. Each experiment was performed at least in triplicate. (**C**) Cells were pre-treated with ROS inhibitors (5 mM NAC or 100 U/ml catalase) for 30 min, and then treated with the indicated treatment. RT-PCR and western blot analysi were employed to evaluate the expression levels of CHOP and DR5. GAPDH was included as loading controls; each experiment was performed at least in triplicate. (**D**) Cells were pre-treated with control/NAC (5 mM)/catalase (100 U/ml)/BAPTA-AM (5 μM)/3-MA (10 mM) before TSN treatment. At 12 h after TSN treatment, cells were harvested and the DR5 surface expression was measured by flow cytometry. Each experiment was performed at least in triplicate. (**E**) A549 cells were pre-incubated with 5 mM NAC or 100 U/ml catalase for 30 min, further treated with TRAIL (100 ng/ml) and TSN (200 nM) for 24 h, the occurrence of apoptotic cells was examined by flow cytometry. Data represent the mean ± SEM from three independent experiments, ***P < 0.001, as compared with control.

Previous studies documented that reactive oxygen species (ROS) play a crucial role in DR5-mediated apoptosis^17^,^18^. Thus, we detected the intracellular H_2_O_2_ and O_2_^−^ levels in A549 cells by using the fluorescent probes 2′,7′-dichlorofluorescin diacetate (DCFH) and dihydroethidium (DHE). As shown in Fig. 5A, TSN triggered the generation of H_2_O_2_ but not of O_2_^−^ in A549 cells. To determine whether the sensitizing effect of TSN was dependent on ROS generation, we pretreated A549 cells with the antioxidants N-acetylcysteine (NAC) and catalase 30 min before TSN treatment. The results demonstrated that TSN-induced H_2_O_2_ generation was attenuated in the presence of NAC or catalase (Fig. 5B). The upregulation of CHOP and DR5 by TSN at both the mRNA (upper panel, Fig. 5C) and protein (lower panel, Fig. 5C) level was significantly abolished in the presence of NAC and catalase (Fig. 5C). NAC and catalase treatment also suppressed the TSN-induced increase in DR5 membrane abundance (Fig. 5D). Accordingly, the cell apoptosis caused by TRAIL plus TSN was significantly attenuated in the presence of NAC or catalase (Fig. 5E).

### Ca^2+^ increase is required for the sensitizing effect of TSN

**Figure 6:**
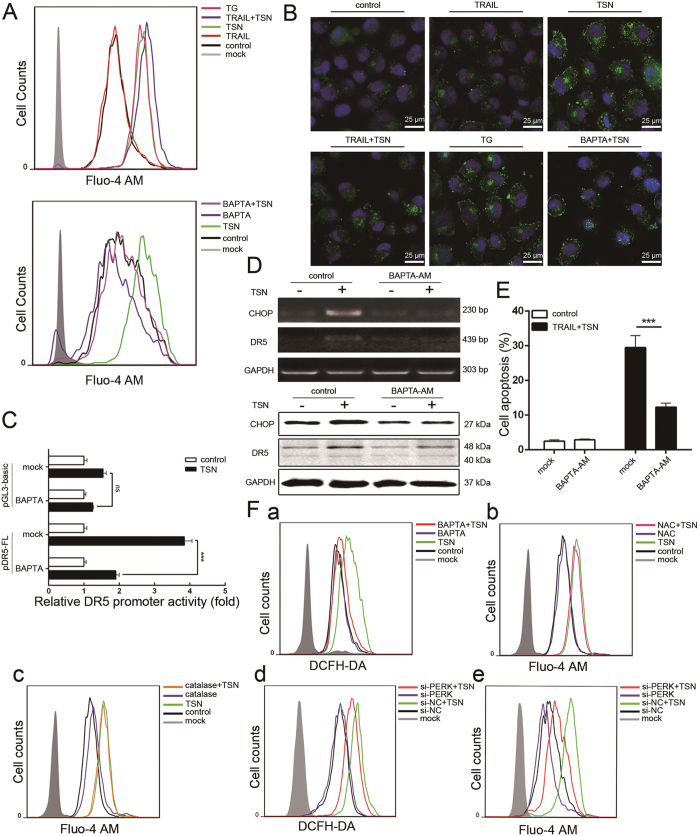
Cytosolic Ca^2+^ is required for the effect of TSN. (**A**,**B**) A549 cells were treated with the indicated treatment for 12 h and stained with 5 μM Fluo-4 AM (Green). The intracellular level of Ca^2+^ was measured by flow cytometry (**A**) and confocal microscopy (**B**). (**C**) A549 cells were transfected with DR5 promoter plasmid for 24 h. After transfection, cells were pre-treated with 5 μM BAPTA-AM for 30 min, and then subjected to TSN treatment. The luciferase activity of untreated samples was arbitrarily set at 1.0 in each group. Data represent the mean ± SEM from three independent experiments. ***P < 0.001, as compared with control. (**D**) A549 cells were pre-treated with Ca^2+^ inhibitors/chelator for 30 min. After TSN treatment, RT-PCR and western blot analysis were employed to evaluate the expression levels of CHOP and DR5. GAPDH was included as loading controls; each experiment was performed at least in triplicate. (**E**) A549 cells were pre-incubated with 5 μM BAPTA-AM for 30 min, then further treated with TRAIL (100 ng/ml) and TSN (200 nM) for 24 h. The occurrence of apoptotic cells was measured by flow cytometry. Data represent the mean ± SEM from three independent experiments, ***P < 0.001, as compared with TRAIL + TSN treatment. (**F**) (a–c) A549 cells were pre-treated with ROS or Ca^2+^ inhibitors/chelator for 30 min before TSN treatment. Then cells were collected and stained with DCFH-DA or Fluo-4-AM for measurement of H_2_O_2_ generation and Ca^2+^ accumulation, respectively. (d,e) A549 cells were transfected with siRNAs for 24 h, and then treated with 200 nM TSN for 12 h. After treatment, cells were collected, stained with DCFH-DA or Fluo-4-AM for measurement of H_2_O_2_ generation and Ca^2+^ accumulation, respectively. Each experiment was performed at least in triplicate.

ROS generation is often accompanied by the disruption of Ca^2+^ homeostasis in cell apoptosis^19^,^20^,^21^. In this study, we evaluated the effect of TSN on intracellular Ca^2+^ levels by using flow cytometry and confocal microscopy analysis. TSN triggered cytosolic Ca^2+^ accumulation in A549 cells when Fluo-4 AM was used as a fluorescent indicator for Ca^2+^ (Fig. 6A, upper panel and Fig. 6B); TG (thapsigargin) served as a positive control. TRAIL treatment, however, had little effect on intracellular Ca^2+^. BAPTA-AM is a chelator of intracellular Ca^2+^. BAPTA-AM treatment significantly suppressed the stimulatory effect of TSN on Ca^2+^ increase (Fig. 6A, lower panel and Fig. 6B), DR5 promoter activation (Fig. 6C), CHOP and DR5 mRNA (upper panel, Fig. 6D) and protein (lower panel, Fig. 6D) expression, and DR5 membrane distribution (Fig. 5D). Under BAPTA-AM treatment, the combined effect of TRAIL and TSN on cell apoptosis was largely attenuated (Fig. 6E). Interestingly, flow cytometry analysis revealed that while TSN-triggered H_2_O_2_ generation could be largely blocked by BAPTA-AM (Fig. 6F-a), antioxidants including NAC (FIG. 6F-b) and catalase (Fig. 6F-c) failed to suppress the TSN-induced Ca^2+^ increase, suggesting that H_2_O_2_ may act downstream of Ca^2+^. It is noteworthy to mention that both H_2_O_2_ generation (Fig. 6F-d) and Ca^2+^ increase (Fig. 6F-e) were impaired by PERK silencing, indicating that ER stress regulates intracellular H_2_O_2_ and Ca^2+^ levels in cells under TSN treatment.

### Autophagy reduces the apoptosis-promoting effect of TSN

**Figure 7:**
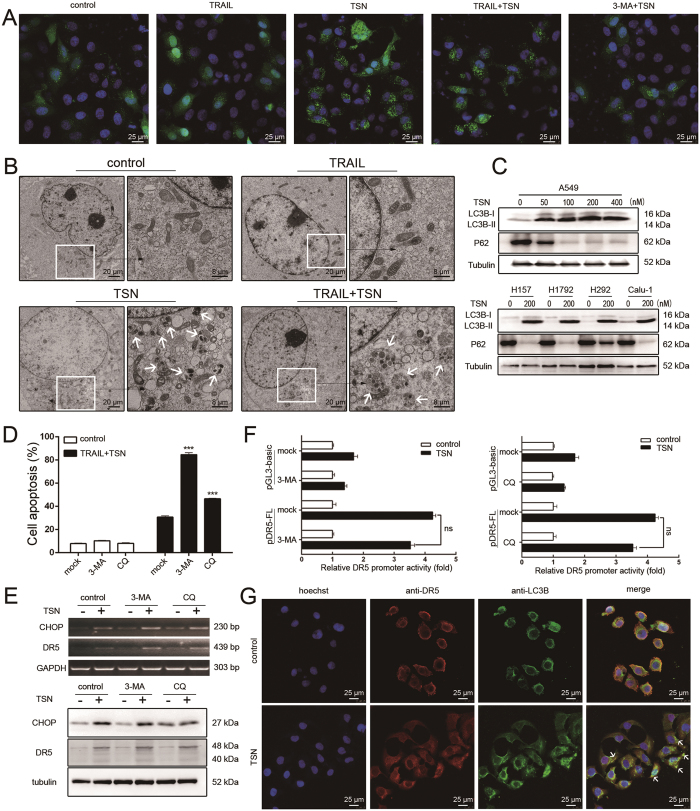
Autophagy alleviates the apoptosis-sensitizing effect of TSN. (**A**) A549 cells were treated with indicated treatment for 8 h, after fixation, cell samples were examined under confocal microscopy. Each experiment was performed at least in triplicate. (**B**) A549 cells were treated with the indicated treatment for 8 h, and then were examined under transmission electron microscopy. (**C**) A549 cells and other NSCLC cells were treated for 12 h with different concentrations of TSN; the total protein was exacted and subjected to western blot analysis to evaluate the level of the indicated proteins. Tubulin was included as loading controls; each experiment was performed at least in triplicate. (**D**) A549 cells were pre-treated with 3-MA or CQ for 30 min, and then treated with 100 ng/ml TRAIL and 200 nM TSN for 24 h. Cell apoptosis was measured by flow cytometry. Data represent the mean ± SEM from three independent experiments, ***P < 0.001, as compared with control. (**E**) A549 cells were pre-treated with 3-MA or CQ for 30 min for 30 min, and further incubated with TRAIL and TSN. RT-PCR and western blot analysis were performed. (**F**) A549 cells were transiently transfected with 0.8 μg DR5 promoter plasmid and 0.2 μg pRL-TK for 24 h, and then incubated with 3-MA or CQ for 30 min before TSN treatment. Promoter activities were measured as previously described. The luciferase activity of untreated samples was arbitrarily set at 1.0 in each group. Data represent the mean ± SEM from three independent experiments. (**G**) A549 cells were treated with 200 nM TSN for 12 h. The autophagosome was visualized by immunostaining of LC3B (green). Hoechst was used to stain nuclei. DR5 was stained with anti-DR5 antibody. The merged pictures are shown in the right panels. White arrows were used to indicate the co-localization of DR5 and LC3B.

It is well-known that ER stress can induce autophagy in cancer cells, a process by which cancer cells survive under stressful conditions^22^. We thus evaluated whether TSN can also induce autophagy in NSCLC cells. As shown in Fig. 7A, confocal microscopy analysis revealed that while many GFP-LC3B dots were observed in TSN or TRAIL/TSN-treated cells, TRAIL treatment alone failed to have an effect, suggesting TSN triggers autophagy in A549 cells. This was further confirmed by the observation that LC3B dots induced by TSN were greatly attenuated in the presence of 3-methyladenine (3-MA), an autophagy inhibitor. Transmission electron microcopy analysis also revealed that many autophagosomes formed in cells after treatment with TSN (Fig. 7B, white arrow). TRAIL treatment alone failed to have an effect (Fig. 7B). The conversion of MAP1LC3B-I to its non-soluble form (MAP1LC3B-II) is considered a hallmark of autophagy. In five human NSCLC cell lines (A549, H157, H1792, H292, and Calu-1), we observed that TSN significantly increased the level of MAP1LC3B-II, while exerting a suppressive effect on p62 expression (Fig. 7C).

To characterize the role of autophagy in the effects of TSN, we pretreated A549 cells with the autophagy inhibitors 3-methyladenine (3-MA) and chloroquine (CQ) before TRAIL plus TSN treatment. Both 3-MA and CQ significantly increased the rate of apoptosis (Fig. 7D), suggesting that autophagy antagonized the apoptosis-sensitizing effect of TSN. These results show that TSN treatment led to both pro-apoptotic and anti-apoptotic effects. To understand the relationship between them, we examined the effects of autophagy inhibitors on CHOP and DR5 expression. Surprisingly, neither 3-MA nor CQ had an effect on TSN-induced CHOP and DR5 upregulation at the mRNA (upper panel, Fig. 7E) or protein level (lower panel, Fig. 7E), or on DR5 promoter activation (Fig. 7F). Nevertheless, flow cytometry analysis revealed that the TSN-induced decrease in membrane DR5 was effectively prevented by 3-MA; 3-MA treatment alone even increased the basal level of membrane DR5 (Fig. 5D), suggesting autophagy may regulate the membrane distribution of DR5. This hypothesis was also confirmed by confocal microscopy analysis (Fig. 7G). Interestingly, in most of the cells subjected to TSN treatment, endogenous DR5 was co-localized with GFP-LC3B. Taken together, we conclude that some membrane DR5 proteins are recruited into autophagy, thus compromising the sensitizing effect of TSN.

### The synergistic anti-tumor effect of TRAIL and TSN *in vivo*

**Figure 8:**
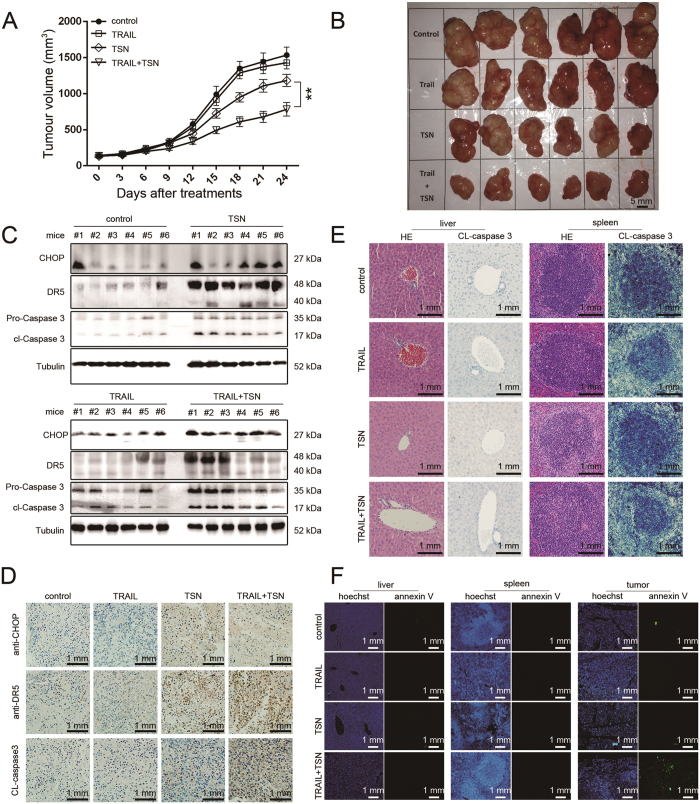
The synergistic anti-tumor effect of TRAIL and TSN *in vivo*. (**A**) To construct xenograft model, A549 cells were inoculated into nude mice through subcutaneous injection. After the average tumor volume reached to 100 mm^3^, the animals were treated with 100 μg TRAIL, 0.173 mg/kg TSN, or both once per day for 24 consecutive days. Tumor volume (**A**) and animal weight (supplementary) were measured once per three days. Data represent the mean ± SEM from three independent experiments, **P < 0.01, as compared with mice treated with TSN alone. (**B**) The solid tumors removed from nude mice. (**C**) After treatment, tumor tissues were removed and lysed in RIPA lysis buffer. Western blot analysis was performed to evaluate the expression levels of the indicated proteins. Similar results were obtained in 3 independent experiments. (**D**) After treatments, tumor tissues were removed and subjected to Immunohistochemistry analysis. The representative image in 3 independent experiments was shown. (**E**) After treatment, the liver and spleen tissues were removed and subjected to H&E staining analysis. The representative image in 3 independent experiments was shown. (**F**) The different tissues removed from nude mice were dewaxed and hydrated, then stained with Hoechst and Annexin V-EGFP. The cell apoptosis was observed under fluorescence microscope. The representative image in 3 independent experiments was shown.

To evaluate the tumor-sensitizing effects of TSN *in vivo*, A549 cells were subcutaneously injected into nude mice to construct a tumor xenograft model. In order to avoid potential hepatotoxicity, animals were given a relatively low concentration of TSN (0.173 mg/kg). The combined therapy significantly slowed tumor growth, but had no evident effects on animal weight (supplementary result Fig. S5). TSN alone had a weak inhibitory effect on tumor growth, while TRAIL alone failed to have an effect (Fig. 8A and B). After treatment for 24 consecutive days, animals were sacrificed and the tumor tissues were removed to prepare samples for western blot, H&E staining, and immunohistochemistry analysis. As shown in Fig. 8C, the protein expression levels of CHOP, DR5, and the cleaved caspase 3 (CL-caspase 3) in animal groups treated with TSN or TRAIL plus TSN were significantly higher than those in animals of the control or TRAIL groups; this result was confirmed by immunohistochemistry analysis (Fig. 8D). Notably, the combination of TRAIL and TSN exhibited low toxicity in tumor-bearing mice, because the cleaved band of caspase 3 was not observed in the liver or spleen tissue of the TSN group or the TRAIL + TSN group (Fig. 8E), as demonstrated by the results of H&E staining and CL-caspase 3 immunohistochemistry analysis. To further examine the toxicity of TSN, annexin V staining was performed on liver, spleen, and tumor tissue. As shown in Fig. 8F, the positive annexin V staining was only seen in tumor tissue isolated from nude mice given TRAIL + TSN treatment. Consistent with the results shown in Fig. 8D, neither TSN alone nor TRAIL + TSN treatment triggered significant cell apoptosis in the mouse liver and spleen. Therefore, TSN selectively increases the sensitivity of tumor cells towards TRAIL-induced apoptosis *in vivo*.

## Discussion

Recombinant soluble human TRAIL, unlike its homologs in the TNF superfamily, has shown selective apoptotic effects on various tumor cells without the risk of lethal systemic inflammation or hepatotoxicity, based on both *in vitro* and *in vivo* experiments^23^. However, the resistance to TRAIL in certain cancer cells has limited its clinical application^24^,^25^,^26^,^27^. Many chemicals or chemotherapeutic drugs with the potential to reverse cancer cell resistance to TRAIL have been discovered; however, few of them can be used in clinical cancer treatment because of their non-selective toxicity in normal tissue. In the present study, we demonstrated for the first time that the natural product TSN has the ability to enhance NSCLC cells’ susceptibility to TRAIL-mediated apoptosis. The concentration of TSN used was too low to cause toxicity to normal cells or tissues. Previous studies have shown that TSN alone has anti-tumor activity^13^. However, the required effective concentration was higher than that used in this study; a higher concentration would have increased the risk of non-specific, toxic side effects. Based on this fact, we presume that TSN is more suitable for use as an adjuvant in cancer treatment to sensitize cells than other therapeutics. In fact, TSN was not only able to sensitize NSCLC cells to TRAIL, but was also able to increase sensitivity to adriamycin at sub-toxic concentrations in breast cancer cells (unpublished observation) and increase sensitivity to a PD-L1 antagonist in melanoma cells (unpublished observation). In addition, TSN was proven to have significant analgesic effects in a variety of diseases, including late-stage cancer. Taken together, use of TSN in clinical cancer treatment warrants further investigation.

Previous studies demonstrated that TSN induced tumor cell death via the mitochondrial pathway; however, we found here that ER stress plays a more prominent role. We presume that this difference in results may be caused by the varying concentrations of TSN used. In this study, ER stress markers including ATF6, IRE1, and GPR78 were upregulated by 100 nM TSN. TSN at a concentration of 50 nM triggered the phosphorylation of PERK, increased CHOP expression, and sensitized A549 cells to TRAIL. However, TSN alone failed to induce cell death, even when it was used at concentrations as high as 400 nM. Therefore, ER stress can be considered an early event that occurs before mitochondrial impairment in NSCLC after TSN treatment. Notably, when NSCLC cells were treated with TRAIL plus TSN, the mitochondrial pathway appeared to be involved because the cleavage activity of pro-caspase 9 was activated. We presume that this mitochondrial impairment is likely not directly caused by TSN treatment, but is a consequence of apoptosis, as reports have shown that both extrinsic and intrinsic pathways are involved in death receptor-mediated apoptosis. Therefore, it is reasonable to conclude that the sensitizing effect of TSN is largely caused by ER stress.

The reason that TSN preferentially enhanced sensitivity to TRAIL-induced apoptosis in NSCLC cells but not in normal cells remains unknown. We presume that this could also be explained by ER stress. It is well-known that ER stress is an adaptive mechanism adopted by cancer cells to survive in the tumor microenvironment^28^. Therefore, cancer cells are likely to be more vulnerable to inducers of ER stress than normal cells. In fact, we did not detect CHOP upregulation in TSN-treated non-cancerous cells.

To evade TRAIL-mediated apoptosis during immune surveillance, cancer cells often downregulate the expression of death receptors. Restoration of death receptor expression is therefore becoming a practical approach to improve the efficacy of TRAIL treatment in cancer cells. Interestingly, although both DR4 and DR5 can bind TRAIL to initiate apoptotic signaling, their roles are not redundant. In this study, TSN was found to increase the expression of both DR4 and DR5; however, only DR5 was involved in augmenting the apoptosis response, as silencing of DR4 had a much lesser effect than silencing DR5. Consistent with this finding, a previous report demonstrated that TRAIL has a higher affinity for DR5 than DR4 in physiological conditions^18^. Similar results were also presented in a study by Kelley *et al*., using phage display^29^.

As a stress-inducible transcription factor, CHOP has both pro-apoptotic and anti-apoptotic functions, and can be rapidly unregulated by a variety of harmful stimuli including UV light, ROS, heat, and ER stress^16^. After activation, nuclear CHOP controls the expression of many genes by acting in concert with other transcriptional factors. In this study, we determined that CHOP is involved in the sensitizing effect of TSN by activating DR5 transcription and expression. It appeared that CHOP upregulation was triggered by ROS generation and Ca^2+^ increase, because antioxidants (NAC and catalase) and BAPTA-AM were able to block TSN-induced CHOP expression. Evidence revealed that ER stress could lead to Ca^2+^ release from the ER to the cytosol^30^. Consistent with these results, we found that TSN-triggered Ca^2+^ increase was largely attenuated in cells transfected with PERK siRNA. The relationship between Ca^2+^ and ROS in apoptotic signaling is rather complex. Depending on the context of stimuli, a Ca^2+^ increase can either act upstream or downstream of ROS generation. Here, we found that TSN-induced ROS generation was suppressed by BAPTA-AM; however, NAC and catalase failed to inhibit the TSN-induced Ca^2+^ increase, and thus, Ca^2+^ acts upstream of ROS. Taken together, ER stress-Ca^2+^ increase-ROS generation-CHOP-DR5 transcription represents the major signaling pathway underlying the sensitizing effect of TSN on NSCLC cells.

The mechanism mediating TSN’s ability to trigger ER stress remains unknown. A previous study demonstrated that TSN was able to increase the conductance of the cell membrane and Ca^2+^ flux in NG108-15 cell lines, which further resulted in the elevation of intercellular Ca^2+^ concentrations^31^, suggesting that TSN may regulate the activity of certain membrane Ca^2+^ channels. Notably, TSN is water-insoluble and has a relatively high molecular weight, indicating that this chemical cannot easily enter the cytosol. Therefore, it is reasonable to presume that there could be a protein receptor specifically recognized by TSN on NSCLC cell membranes, and that this receptor could be a membrane Ca^2+^ channel. By binding to this putative receptor, TSN induces the ER stress response, thereby increasing NSCLC cell sensitivity to TRAIL. However, this hypothesis requires more experiments for verification.

ROS have a dual role in regulating apoptosis. In this study, the pro-apoptotic role of ROS in TSN-treated NSCLC cells was predominant. ROS mainly consist of H_2_O_2_ and O_2_^−^. There are two major sources of intracellular ROS: membrane NADP/NADPH oxidase and mitochondria. Membrane NADP/NADPH oxidase mainly generates O_2_^−^, whereas both H_2_O_2_ and O_2_^−^ can be produced by the mitochondria. In this study, H_2_O_2_ was mainly responsible for the sensitizing effect of TSN, suggesting it may be produced in the mitochondria. Interestingly, reports have shown that the ER and mitochondria are physically and functionally conterminous, and the ER-stress sensor PERK is thought to be a crucial element of the mitochondria-associated ER membranes (MAMs), linking these two organelles^32^. MAMs are critical in the regulation of Ca^2+^ dynamics and the passive discharge of Ca^2+^ from the ER^32^. Thus, PERK most likely couples Ca^2+^ increases and ROS generation in TSN-treated NSCLC cells through ER-mitochondria crosstalk.

Besides ER stress, TSN also triggered autophagy in NSCLC cells. Previous studies revealed that an increase in cytosolic Ca^2+^ can activate a series of enzymes that are possibly associated with autophagy signaling^22^. Autophagy can also be induced by inhibition of AKT1 activity through the ATF4-CHOP-TRIB3 pathway following ER stress^33^. Based on these facts, it is reasonable to presume that TSN-induced autophagy is likely mediated by ER stress and Ca^2+^ signaling. Interestingly, TRAIL has been reported to induce autophagosome formation in a variety of cell lines^34^,^35^,^36^,^37^, and endogenous TRAIL is essential for lumen formation during autophagy^37^. However, we did not observe autophagy in NSCLC cells after TRAIL treatment in this study. Thus, TRAIL-induced autophagy is likely cell type-dependent. Previous studies demonstrated that autophagy modulated resistance to TRAIL in tumor cells^38^,^39^,^40^,^41^ via multiple mechanisms, including degradation of caspase 8^35^, inadequate formation of DISC^41^, cleavage of ATG6^40^, activation of the NF-κB pathway^39^, and degradation of P62^38^. In this study, we identified a novel role for autophagy in modulating tumor sensitivity to TRAIL; that is, autophagy recruits membrane DR5 and counteracts TRAIL-mediated apoptosis.

Interestingly, although ER stress and autophagy are intricately linked, they exerted contrasting effects on modulation of DR5 expression in this study. ER stress transcriptionally upregulated DR5 gene expression, while autophagy decreased DR5 membrane abundance post-translationally. To explain this inconsistency, we presume that autophagy is likely to be a negative feedback mechanism adopted by NSCLC cells to react against stress caused by TSN. If this is true, it is possible that while TSN was able to overcome NSCLC cells’ resistance to TRAIL, NSCLC cells in turn developed resistance to TSN by autophagy induction. Stress response plays complicated roles in cancer development and treatment. Cancer cells can rapidly respond to therapeutic interventions and decrease the initial effectiveness of treatment by gradually developing drug resistance mechanisms. In order to achieve long-lasting anti-tumor efficacy, it is absolutely necessary to regularly monitor the status of drug resistance in clinical cancer treatment.

## Materials and Methods

### Reagents

Human recombinant TRAIL was prepared as previously described^10^. Dulbecco’s modified Eagle’s medium (DMEM) and RPMI 1640 medium were from Wisent (Wisent, St-Bruno, Quebec, Canada). Lipofectamine 2000 was from Invitrogen (Carlsbad, CA, USA). Caspase 3 inhibitor (AC-DEVD-CHO), caspase 8 inhibitor (Z-IETD-FMK) and caspase 9 inhibitor (Z-LEHD-FMK) were from Calbiochem (San Diego, CA, USA). H_2_DCFDA and DHE fluorescent dyes were purchased from Molecular Probes (Eugene, OR, USA). Actinomycin D (Act D), N-acetyl-L-cysteine (NAC) and catalase were from Sigma (St. Louis, MO, USA). Fluo-4-acetoxymethyl esterM (Fluo-4 AM) was from Dojindo (Kumamoto, Japan). BAPTA-AM, 3-MA and chloroquine were from Selleck Chemicals (Houston, TX, USA).

### Clinical sample collection

The heparinized blood in our study was obtained from healthy adult donors (Department of Anesthesia, Changhai Hospital, affiliated hospital of the Second Military Medical University). The Non-small cell lung carcinoma samples in this project were from Jiangsu Cancer Hospital. All subjects enrolled in this study fulfilled the criteria defined by the 2001 International Sepsis Definitions Conference. The experiment involving human subject was approved by the Medical Ethics Committee of Changhai Hospital of China and the Medical Ethics Committee of Jiangsu Cancer Hospital of China. The informed consent was obtained from all patients and healthy donors involved in this project, and the experiments conformed to the principles set out in the WMA Declaration of Helsinki and the Department of Health and Human Services Belmont Report.

### Cell lines and cell culture

All NSCLC cell lines and normal cells used in this study were original from the American Type Culture Collection (ATCC, Manassas, VA, USA). LAC521 cells (lung adenocarcinoma 521) were provided by Dr. Lin Ma in our group, and the cells were isolated from clinical lung cancer sample following conventional protocols. Human peripheral blood mononuclear cells (PBMC) were isolated from heparinized blood of healthy adult donors following Ficoll/Paque (Pharmacia, Uppsala, Sweden) density gradient centrifugation method. Cells were cultured in DMEM (A549, H292, SW1573, 293 T, L02) or PRMI 1640 (H157, H1792, Calu-1, H460, H1299, LAC521, Beas-2B, HBE, PBMC) medium, which contain 10% (v/v) fetal bovine serum (FBS) and 50 U/ml penicillin/streptomycin. Cells were maintained at 37 °C in a humidified incubator with 95% air and 5% CO2. When the confluence reached to a certain extent, cells will be exposed to different treatment. The details of treatment are stated in figure legends.

### Western blot analysis

Western blot analysis was performed as previously described^42^. DR4 antibody was from Novus (Novus Biologicals, Littleton, CO, USA); DR5 and P62 antibodies were from Abcam (Cambridge, UK); FLIP-L and FLIP-S antibodies were purchased from Santa Cruz Biotechnology (Santa Cruz, CA, USA); Caspase 3, Caspase 8, Caspase 9, PARP, CHOP, PERK, p-GCN2, GCN2, p-PKR, PKR, HRI, p-eIF2α, eIF2α, ATF4, MAP1LC3B, tubulin, GAPDH and β-actin antibodies were from Cell Signaling Technology (Beverly, MA, USA); The secondary antibodies used were HRP-conjugated anti-mouse IgG and anti-rabbit IgG from sigma (USA).

### Plasmid construction, transient transfection and luciferase assays

Renilla luciferase and firefly luciferase constructs were obtained from Promega (Madison, WI, USA). To create the plasmid containing human DR5 promoter sequence, the 5-flanking region of human DR5 genomic DNA was amplified by PCR from A549 genomic DNA and cloned into pGL3 firefly luciferase reporter vector with Kpn I (Thermo Scientific, Hudson, NH, USA) and Bgl II (Thermo Scientific, Hudson, NH, USA) sites. The deletion mutants and point mutations in the Elk-1-, CHOP-, and NF-κB-like sites were generated by a two-step PCR method^43^. Other expressing plasmids including EGFP-LC3B, full length pGl3-DR4 promoter and its deleted variants were all generated in our lab by RT-PCR and molecular cloning methods. These plasmids were confirmed by DNA sequencing and transfected into cells by polyJet (Signagen Laboratories, Ijamsville, MD, USA) transfection reagent according to manufacturer’s instruction.

For luciferase assays, cells were harvested and lysed in 1× lysis buffer after transfection and treatment. Promoter activities were measured by the Dual Luciferase Reporter Assay System (Promega, Madison, WI, USA) following the manufacturers’ instructions. The relative luciferase activity represents the ratio of firefly luciferase to renilla luciferase.

### Quantitative RT-PCR and Semiquantitative RT-PCR

Total RNA was exacted from intact cells by using TRIZOL agent and chloroform according to the seller’s RNA extraction protocol. For reverse transcription, equal amount of total RNA were used following the manufacturer’s instructions (Toyobo, Osaka, Japan). Quantitative RT-PCR (qRT-PCR) was performed as previously described^42^. The PCR primers employed to amplify ATF6, IRE1, GPR78, CHOP, DR5, DR4, GAPDH and tubulin were listed as follows:

ATF6: sense, 5′-ACACAGCTCCCTAATCACGT-3′, antisense: 5′-TTTAATCTCGCCTCTAACCC-3′;

IRE1: sense, 5′-CGGGAGAACATCACTGTCCC-3′, antisense, 5′-CCCGGTAGTGGTGCTTCTTA-3′;

GRP78: sense, 5′-GAAGATTGGCATTGCTATTG-3′, antisense, 5′-TGTGTCCACAGAGCCGTTGT-3′;

CHOP: sense, 5′-CCCTCACTCTCCAGATTCC-3′, antisense, 5′-GAGTCGCCTCTACTTCCCT-3′;

DR5: sense, 5′-ACTCCTGGAATGACTACCTG-3′, antisense, 5′-ATCCCAAGTGAACTTGAGCC-3′;

DR4: sense, 5′-TTGTGTCCACCAGGATCTCA-3′, antisense, 5′-GTCACTCCAGGGCGTACAAT-3′;

GAPDH: sense, 5′-CACCATCTTCCAGGAGCGAG-3′, antisense, 5′-ATGAGTCCTTCCACGATACC-3′;

DR5 (qRT-PCR): Sense, 5′-CCAGCAAATGAAGGTGATCC3′, Antisense, 5′-GCACCAAGTCTGCAAAGTCA3′;

tubulin (qRT-PCR): Sense, 5′-CCCCTTCAAGTTCTAGTCATGC-3′, Antisense, 5′-ATTGCCAATCTGGACACCA-3′;

### Measurement of apoptotic cells by Annexin V/PI double staining

The measurement of cell apoptosis by Annexin V/PI double staining was performed following previously illustrated method^11^, briefly, cells after treatment were harvested and stained with 1 ug/ml Annexin V-EGFP in ice-cold binding buffer for at least 15 min. Right before analysis by flow cytometer, PI was added to each sample. FL1 channel and FL3 channel were used to measure EGFP and PI fluorescence emission, respectively. Cell doublets were excluded through fluorescent compensation. A total of ten thousands cells were countered per sample.

### RNA interference

A549 cells were transfected with the siRNA against CHOP, DR4, DR5, PERK, GCN2, HRI and PKR using lipofectamine 2000 (Invitrogen, Carlsbad, CA, USA). Twenty-four hours after transfection, cells were exposed to the indicated treatment. The silencing effect of siRNAs was evaluated through RT-PCR or western blot.

### Chromatin immunoprecipitation

Chromatin immunoprecipitation assay was performed as previously described^42^. The primers sequence we used here are: Sense, 5′-AGGTTAGTTCCGGTCCCTTC-3′; and anti-sense, 5′-CAACTGCAAATTCCACCACA-3′ (reverse), which amplify a 111 bp region of the human DR5 promoter containing a CHOP binding site: GAGGATTGCGTTG^44^.

### Measurement of surface expression of death receptors

Measurement of surface expression of death receptors was performed as previous described^10^. Briefly, A549 cells were treated as indicated for 12 h, then cells were collected and incubated with DR4 or DR5 antibody for 2 h at 4 °C. Subsequently, cells were dyed with Alexa Fluor^®^ 488 conjugated secondary antibodies (Invitrogen, Carlsbad, CA, USA) and analyzed by flow cytometry in FL1 channel.

### Measurement of intercellular ROS generation

The level of intercellular ROS generation was evaluated by staining cells with H_2_DCFDA and DHE as previously detailed^10^. To be specific, cells after treatment were incubated with 10 μM H_2_DCFDA or 5 μM DHE to detect the generation of H_2_O_2_ and O_2_^−^ by flow cytometry in FL1 and FL3 channels, respectively.

### Measurement of cytosolic Ca^2+^ level by flow cytometry

Measurement of cytosolic Ca^2+^ level by flow cytometry was conducted following the conventional procedures^45^. Briefly, A549 cells were grown in 12 well-plate to 40–60% confluence before indicated treatment. After treatment for 12 h, cells were washed and stained with 5 μM Fluo-4 AM following the manufacturers’ instructions. The intercellular Ca^2+^ level was measure in FL1 channel by using flow cytometry.

### Measurement of cytosolic Ca^2+^ level by confocal microscopy

A549 cells were grown to 40% confluence on a chamber slide and treated with drugs or chemicals for 12 h. After treatment, the chamber slide (with cells) were washed with ice-cold PBS and stained with 5 μM Fluo-4 AM for 30 min at 37 °C. Then cells were washed with PBS and fixed with 4% paraformaldehyde for 10 min. After that, the cells were mounted with glycerin on a glass slide and observed under confocal microscope.

### Immunofluorescence microscopy

For co-location of DR5 with LC3B, A549 cells were grown to 40% confluence on a chamber slide and treated for 12 h. Then cells were washed with ice-cold PBS and fixed with 4% paraformaldehyde for 30 min. After incubated with 1% Triton for 30 min and blockage for 1 h, cells were incubated with LC3B (mouse) and DR5 (rabbit) antibodies for 2 h at 37 °C or overnight at 4 °C. Secondary antibodies Alexa Fluor^®^ 488 (Invitrogen, Carlsbad, CA, USA) and Alexa Fluor^®^ 594 (Invitrogen, Carlsbad, CA, USA) were employed to distinguish DR5 and LC3B under confocal microscope at 488 nm and 594 nm wave length. Subsequently, cells were washed and stained with Hoechst for 5 min, the wave length for Hoechst detection was 405 nm.

For observation of accumulated autophagosome, A549 cells were grown on a chamber slide to 40% confluence and transiently transfected with 1 μg EGFP-LC3B plasmid. After 24 h transfection, cells were challenged with indicated treatment for 8 h and stained with Hoechst as above mentioned. EGFP-LC3B was observed at 488 nm using confocal microscope.

For staining of tissues with Annexin V, paraffin sections were dewaxed and hydrated, then washed two times with 1X binding buffer and stained with 1 μg/ml Annexin V-EGFP which was diluted in binding buffer for 30 min. Subsequently, sections were stained with Hoechst for 5 min and observed under fluorescence microscope.

### Transmission electron microscopy (TEM) analysis

Transmission electron microscopy (TEM) was performed to capture autophagic vacuoles following the conventional protocols described previously^30^. For cell treatment, A549 cells were grown in 10-cm dishes to 70% confluence and treated with or without TRAIL or TSN as indicated for 8 hours, and then cells were collected and washed with ice-cold PBS. After centrifugation, cell cluster was fixed in 4% glutaraldehyde.

### Xenograft tumor models and drug treatment *in vivo*

The animal experimental protocols were approved by the Animal Care and Protection Committee of Nanjing University-Gulou hospital (SYXK 2004-0013). The authors confirmed that all animals received human care and all animal experiments were performed in accordance with the relevant guidelines and regulations. The animals were housed in standard cages at 25 °C, on a 12/12 light–dark cycle in a clean room, and they were supplied with food and water ad libitum. Construction of xenograft models was performed according to methods as previously described^10^. Once tumor volume reached 100 mm^3^, animals were randomly assigned into four groups and the mean value of starting tumor volume was similar. Tumor volume was calculated by the ellipsoid formula: 1/2*D*d^2^, in which ‘D’ stands for the longest diameter of the tumor, and ‘d’ represents the smallest diameter. For drug delivery, animals were injected intraperitoneally with 0.173 mg/kg TSN, or 100 μg TRAIL, or their combination once per day for 24 consecutive days. Tumor volume and animals weight were measured once per three days. At the end of the experiments, animals were killed and the tumors, livers, and spleens were removed to prepare paraffin sections for H&E staining and Immunohistochemistry.

### Immunohistochemistry analysis

As above mentioned, paraffin sections from xenograft models were dewaxed and hydrated before antigen retrieval. Then sections were incubated with CHOP antibody or cleaved caspase 3 antibody or DR5 antibody for 2 h at 37 °C, or overnight at 4 °C. Then sections were incubated with HRP conjugated secondary antibodies for 1 h. Signals were developed using liquid diaminobenzidinetetrahydrochloride (Gene tech, Shanghai, China) following the manufacturer’s instructions.

### Statistical analysis

Data are presented in the figure as mean ± SEM. The significance of differences between two groups was determined by two-tailed Student’s *t*-test. Multiple comparisons were made with analysis of variance (ANOVA) followed by Bonferroni’s *post hoc* test. For all statistical analysis, GraphPad Prism 5 software for Windows was used (GraphPad Software, San Diego, CA).

## Additional Information

**How to cite this article**: Li, X. *et al*. Reversal of the Apoptotic Resistance of Non-Small-Cell Lung Carcinoma towards TRAIL by Natural Product Toosendanin. *Sci. Rep.* **7**, 42748; doi: 10.1038/srep42748 (2017).

**Publisher's note:** Springer Nature remains neutral with regard to jurisdictional claims in published maps and institutional affiliations.

## Supplementary Material

Supplementary Results
