# Supplementary material for: Reversal of the Apoptotic Resistance of Non-Small-Cell Lung Carcinoma towards TRAIL by Natural Product Toosendanin
Source: Sci Rep. 2017 Feb 17;7:42748. doi: 10.1038/srep42748 (PMC5314365; doi:10.1038/srep42748)
Supplement: Supplementary Results [file srep42748-s1.pdf]

Reversal of the Apoptotic Resistance of Non-Small-Cell Lung Carcinoma towards TRAIL by Natural Product Toosendanin

Xin Li, Ming You, Yong-jian Liu, Lin Ma, Pei-pei Jin, Ri Zhou, Zhao-Xin Zhang, Baojin Hua,

Xiao-jun Ji, Xiao-ying Cheng, Fangzhou Yin, Yan Chen, and Wu Yin

Supplementary results

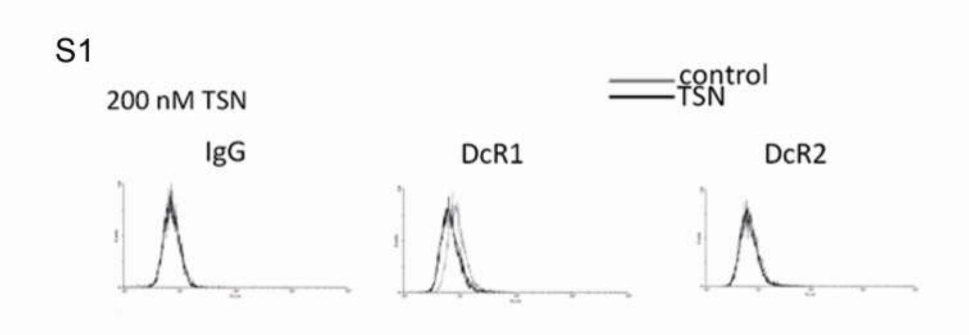

FIG. S1. Effect of TSN on the surface expression of decoy TRAIL receptors. The surface expression of decoy TRAIL receptors (DcR1 and DcR2) was examined by flow cytometry. A549 cells were treated with 200 nM TSN, after treatments, cells were collected and incubated with rabbit normal IgG, anti-DcR1, and anti-DcR2 antibodies separately (Bioss, Beijing, China). The Alexa Fluor488 conjugated goat-anti-rabbit IgG was used as the second antibody. Data were analyzed using WinMDI2.9 software. Similar results were obtained in 3 independent experiments.

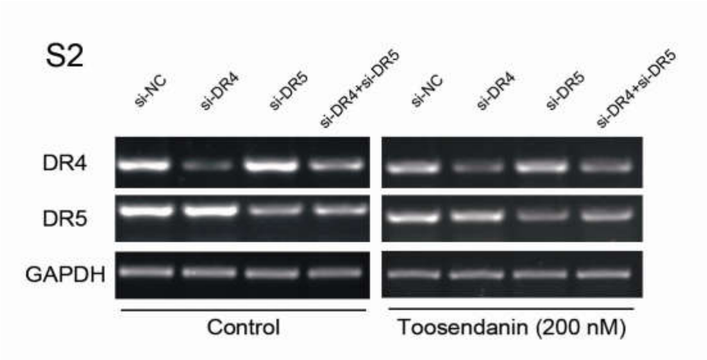

Fig. S2. Silencing of the DR4 and DR5 expressions in A549 cells. Cells were transfected with the indicated siRNAs for 24 h, and then treated with or without 200 nm TSN for 12 h. After treatment, cells were collected and RT-PCR was performed to measure the mRNA expression level of death receptors.

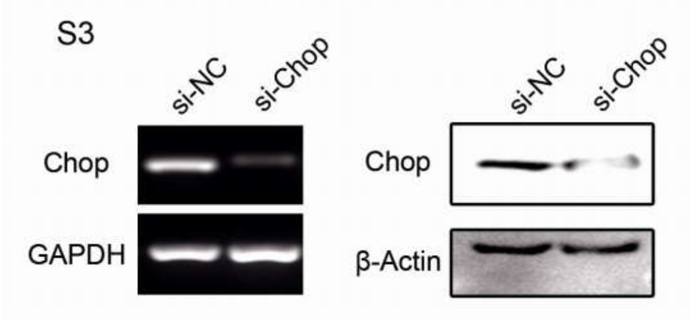

Fig. S3. Silencing of the CHOP expression in A549 cells. Cells were transfected with the indicated siRNAs for 24 h. Then cells were collected, RT-PCR and western blot analysis were performed to measure the expression level of CHOP.

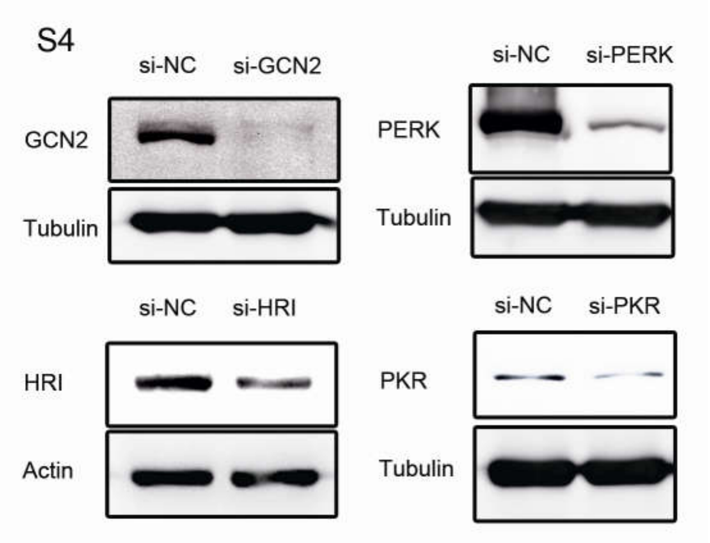

Fig. S4. Silencing of the eIF2-α kinases expression, including GCN2, PERK, HRI, and PKR in A549 cells. Cells were transfected with the indicated siRNAs for 24 h. Then cells were collected, western blot analysis was performed to measure the expression levels of eIF2α kinases.

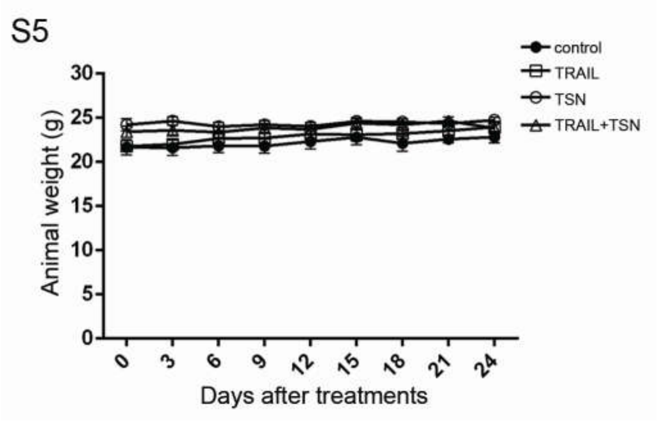

Fig. S5. Effects of TRAIL and TSN on body weight of A549 xenograft nude mice. Cells were inoculated into nude mice through subcutaneous injection. Animals were treated with 100 µg TRAIL, 0.173 mg/kg TSN, or both once per day for 24 consecutive days after the average tumor volume reached to 100 mm^3. Animal weight was measured

once per 3 days, n=6.
